# Neophobia and social facilitation in narrow-striped mongooses

**DOI:** 10.1007/s10071-020-01429-5

**Published:** 2020-09-21

**Authors:** Bako N. Rasolofoniaina, Peter M. Kappeler, Claudia Fichtel

**Affiliations:** 1 Dept. Sociobiology/Anthropology, Johann-Friedrich-Blumenbach Institute of Zoology and Anthropology, University Göttingen, Göttingen, Germany; 2 Behavioral Ecology and Sociobiology Unit, German Primate Center Leibniz Institute for Primate Research, Göttingen, Germany

**Keywords:** Carnivora, Social facilitation, Neophobia, Attendance bias, *Mungotictis decemlineata*

## Abstract

Social learning is widespread in the animal kingdom, but individuals can differ in how they acquire and use social information. Personality traits, such as neophobia, may, for example, promote individual learning strategies. Here, we contribute comparative data on social learning strategies in carnivorans by examining whether narrow-striped mongooses (*Mungotictis decemlineata*), a group-living Malagasy euplerid, learn socially and whether neophobia influences social learning. To this end, we tested seven wild female groups with a two-option artificial feeding box, using a demonstrator–observer paradigm, and conducted novel object tests to assess neophobia. In five groups, one individual was trained as a demonstrator displaying one of the techniques, whereas the other two groups served as control groups. Neophobia did not co-vary with an individual’s propensity to seek social information. However, less neophobic individuals, and individuals that tended to seek social information, learned the task faster. Moreover, individuals in demonstrator groups learned the task faster than those in groups without a demonstrator and used the demonstrated technique more often. Hence, narrow-striped mongooses rely on social facilitation and local or stimulus enhancement to solve new problems. Finally, our results suggest that several individual characteristics should be taken into consideration to obtain a more comprehensive understanding of social learning strategies.

## Introduction

Learning by observing others is a mechanism for behavioural plasticity that can shape the behavioural repertoire of an individual (Kendal et al. 2010; Aplin 2016). Social learning is associated with many benefits, from acquiring behavioural traits in different contexts to the establishment of cultural behaviour across populations (Aplin 2016; Whiten and van de Waal 2018). However, social learning also comes with costs, as it can spread incorrect information (Rieucau and Giraldeau 2011). Consequently, individuals should ideally switch flexibly between asocial and social learning strategies (Kendal et al. 2018). To maximize the benefits, an individual should flexibly use social learning strategies to decide from whom and when to learn, and which information to acquire (Laland 2004; Kendal et al. 2018). Social learning strategies encompass social information seeking and its use or application in future contexts. For instance, one type of a social learning strategy, the “state-based strategy”, depends on the observer’s intrinsic characteristics, such as rank, age, and sex, which may influence its decision to learn socially in a given situation (Kendal et al. 2018). Moreover, a state-based strategy will influence an individual’s choice to be attentive to a conspecific to seek social information, triggering a preferential attendance bias (Kendal et al. 2018), resulting in directed social learning or a transmission bias (Kendal et al. 2015). As a result, the tendency to seek and use social information can vary at the individual level across group members (Mesoudi et al. 2016; Watson et al. 2018).

One of the main factors driving this variation is animal personality, i.e., individual differences in behaviour that are consistent across time and contexts (Réale et al. 2007; Kurvers et al. 2010a; Mesoudi et al. 2016). Personality traits, such as boldness, neophobia or exploration, appear to influence the tendency to use social information and provide a benefit against danger in a high-risk environment (Greggor et al. 2015; Crane and Ferrari 2017). For example, in a social foraging experiment, shyer barnacle geese (*Branta leucopsis*) were more likely to use social information than their bolder conspecifics because they had fewer opportunities to gather individual information and, hence, relied more on conspecific demonstrators to find high-quality food patches (Kurvers et al. 2010a). In contrast, in great tits (*Parus major*), bolder individuals were more inclined to profit from social learning because the more fearless individuals hindered shyer individuals from participating in the learning situation (Marchetti and Drent 2000). Furthermore, neophobia can also be transmitted socially as for example in fathead minnows (*Pimephales promelas*), where naïve observers also learned neophobic responses from a demonstrator in a social learning paradigm (Crane et al. 2015).

Exploration influenced social learning in a study examining mate choice and food choice, with less explorative female zebra finches (*Taeniopygia guttata*) copying the decision exhibited by a conspecific model even if it would lead to unfavourable choices, such as the choice of a non-preferred male or non-preferred food (Rosa et al. 2012). Although female zebra finches were previously tested for their individual preferences, they still followed the model’s choice, indicating that they prioritised social information over personal information (Rosa et al. 2012). In three-spined sticklebacks (*Gasterosteus aculeatus*), more explorative individuals were more likely to join an unfamiliar demonstrator because less explorative individuals needed more time to familiarize themselves with the new conspecific and were, hence, more sensitive to risk (Nomakuchi et al. 2009). Therefore, personality traits can modify the tendency to learn socially by obtaining access to either personal or social information, as well as the tendency to rely more on social learning in contexts where unfamiliar objects/conspecifics are involved. However, whereas the effect of exploratory behaviours on social learning is well known (summarised in Mesoudi et al. 2016), the effect of neophobia on the tendency to seek social information remains obscure in comparison.

Although social learning is widespread across animals (insects: Slaa et al. 2003; Grüter and Leadbeater 2014; fish: Nomakuchi et al. 2009; Webster and Laland 2017; birds: Marchetti and Drent 2000; Morales Picard et al. 2017; carnivorans: Thornton and Clutton-Brock 2011; primates: Schnoell and Fichtel 2012; van de Waal et al. 2013), it has been less often studied experimentally in social carnivores. For instance, spotted hyenas (*Crocuta crocuta*) did not learn socially in a problem-solving task (Benson-Amram et al. 2014). For meerkats (*Suricata suricatta*), however, individuals preferentially chose the landmark in a two-choice task that was also preferred by a demonstrator, indicating inadvertent social learning via stimulus enhancement (Thornton and Malapert 2009). Teaching, a highly derived form of social learning, has been shown in meerkats, with adults providing pups opportunities to interact with live prey to learn prey-handling skills (Thornton and McAuliffe 2006). Moreover, young banded mongooses (*Mungos mungo*) imitate the foraging technique exhibited by adult individuals (Müller and Cant 2010). Thus, patterns of social learning appear to be highly variable across carnivorans.

To contribute new comparative data to this field of research, we investigated the presence of social learning in narrow-striped mongooses (*Mungotictis decemlineata*). Specifically, we examined the influence of personality on an individual’s probability to learn socially in a social diffusion task. Female narrow-striped mongooses live in stable, hierarchical groups (3.7 ± 0.4 individuals) (Schneider and Kappeler 2016), and exhibit a generalist and opportunistic feeding ecology (Rasolofoniaina et al. 2019). Group members regularly forage for hidden prey, which may present opportunities for social learning to acquire relevant hunting strategies, exploration of novel food, or space use. In particular, we examined whether an individual’s tendency to learn socially is related to individual variation in neophobia, because neophobia is generally thought to hinder individual learning (Webster and Lefebvre 2001).

Using field experiments, we examined individual variation in neophobia by presenting novel objects, and we studied social learning by conducting a social diffusion experiment. Social diffusion experiments are set out to study how founder behaviours spread across multiple individuals in a group (Whiten and Mesoudi 2008). By presenting an artificial feeding apparatus that can be opened by two different techniques and for which demonstrators have been trained to use only one of the two techniques, social learning has been demonstrated in various species (primates: Pesendorfer et al. 2009; van de Waal et al. 2010; Schnoell and Fichtel 2012; Claidière et al. 2013; birds: Morales Picard et al. 2017). To investigate experimentally social learning, we confronted narrow-striped mongooses with such a two-option feeding apparatus. We predicted that: (1) if neophobia positively influences the propensity to observe the demonstrator, more neophobic individuals are expected to spend more time with the demonstrator manipulating the task than less neophobic individuals, (2) If neophobia positively influences social learning, less neophobic individuals are expected to learn the task faster than more neophobic individuals, (3) If narrow-striped mongooses learn socially, we predicted that the presence of a demonstrator should improve the learning speed of observers compared to individuals learning without demonstrator, and (4) individuals are more likely to use the demonstrated technique to open the feeding appartus.

## Methods

### Study animals and general testing procedure

Between November 2014 and September 2017, we studied seven female groups (Table 1) from an individually-marked population of free-ranging narrow-striped mongooses (Schneider and Kappeler 2016) in Kirindy Forest, Madagascar. Individuals in a group were marked either by radio-collars or with specific fur-shaving patterns on the tail. Groups were located within their territory using radio-tracking and were tested opportunistically. For testing, we used the following general experimental procedure: apparatuses were baited with dry cat food out of sight of the individuals, and animals were lured with an acoustic signal, shaking a plastic box containing cat food to the experimental area (Schnoell and Fichtel 2012). The experiment started when the first individual of the group approached one of the apparatuses within a range of 3 m and ended when the last individual left the arena.

**Table 1:** Group composition (all female adults and the juveniles and infants of unknown sex) and experimental condition during the social learning task

Group	Number of individuals	Condition
B	3 (3 adults)	Control
L	3 (2 adults and 1 juvenile)	Control
C	6 (4 adults and 2 juveniles)	Pull
N	4 (2 adults, 1 juvenile and 1 infant)	Pull
G1	2 (1 adult and 1 infant)	Pull
L1	2 (2 adults)	Slide
M	5 (5 adults)	Slide

### Novel object test

Neophobia was assessed by presenting a novel object next to a wooden plate containing food. Both objects were placed in the middle of a metal ring, allowing an accurate estimate of the distance between the subject and the novel object. Individuals were previously habituated to the metal ring before conducting the novel object test. To avoid monopolisation by certain individuals, we presented one experimental set-up per individual. To assess the repeatability of the personality trait “neophobia,” we conducted two novel object tests, by presenting either colourful plastic balls or red plastic cups as novel objects. In six groups, we repeated the novel object test after a period of 3 years, whereas in one group, marked at the end of the study, we repeated the novel object test after a period of 4 months. The average time between the two novel object tests was 12.5 ± 12.02 (mean ± SD) months. We tested 33 individuals in the first novel object test but only 15 individuals in the second novel object test, due to individual losses over the 3 years or a lack of motivation of some individuals to approach the experimental area.

Based on video-recordings, we measured the following behaviours from the two novel object tests: latency to enter the metal ring, latency to contact the novel object, and latency to feed next to the novel object. We estimated the repeatability of each individual latency with individuals that participated in both novel object tests (*N* = 15), using the package “rptR” (Nakagawa and Schielzeth 2010). Before the analyses, we log-transformed the variables to achieve normality. We computed point estimates of repeatability *R*, *p* values, standard errors SE, and the confidence intervals with bootstrapping. The significant repeatable latency to feed next to the novel object was retained and defined as “neophobia.” Individuals exhibiting longer latencies were categorised as more neophobic, whereas those with shorter latencies were the less neophobic ones.

### Social learning experimental set-up

**Fig. 1:**
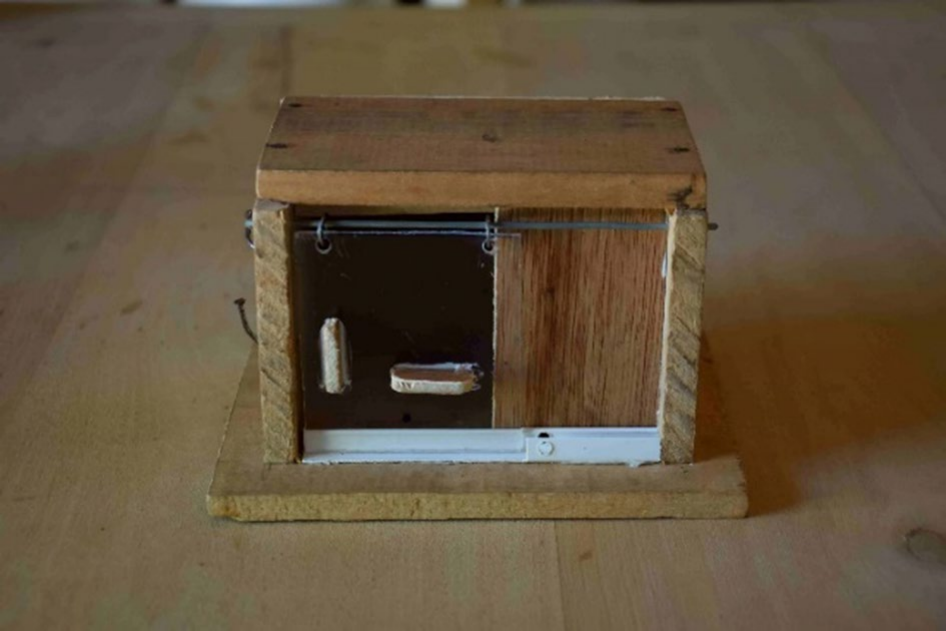
Two-option foraging apparatus used to assess social learning. The door can be opened by either sliding or pulling

A problem-solving feeding apparatus (Fig. 1) was constructed similar to an apparatus that has been used in a study of social learning in vervet monkeys (*Chlorocebus aethiops*) (van de Waal and Bshary 2011). The apparatus consisted of a wooden box (9.5 cm × 13.6 cm), with a transparent plexiglass door, fixed on a wooden plate (13 cm × 17 cm). The door could be opened via two opening mechanisms, by either pulling or sliding the plexiglass door. We first trained a demonstrator by presenting only one box that could be opened by one technique only. Following this training, we conducted the group experiment by presenting several boxes that could be opened by both techniques and we presented one box for each group member. In total, we tested seven groups: five groups in which a demonstrator was trained to open the box with either the pull (*N* = 3 groups) or the slide technique (*N* = 2 groups), and two groups in which no demonstrator was trained served as control groups. For the control groups (*N* = 2), we used boxes that could be opened by both techniques.

### Demonstrator training procedure

To train the demonstrator, only one feeding apparatus was presented, which was monopolized across sessions and groups by the oldest and dominant female of a group, and she served as the demonstrator (Schneider and Kappeler 2016). In three groups, we trained the demonstrator to open the apparatus with the pull technique by blocking the slide technique, whereas in the other two groups, we trained the demonstrator to open the apparatus with the slide technique. For the training, we presented the boxes once per day and the demonstrator could perform only one trial over the course of 20 days, resulting in 20 trials performed by the demonstrator. To ensure that they had learned the task, each demonstrator had to open the box more often than expected by chance by reaching a learning criterion of 80% successful trials.

Since groups of narrow-striped mongooses always forage together (Schneider and Kappeler 2016), other group members (observers) were present in the experimental arena and could approach the demonstrator while she manipulated the apparatus. To assess the propensity to seek social information, we placed a metal ring (50 cm diameter, the same metal ring used during the novel object tests) around the feeding apparatus during the presentation, allowing us to measure the time spent close to the demonstrator for each observer. To prevent observers from manipulating the boxes during the training of demonstrators, the hinge of the door was built tight, so that it stayed open and did not close automatically after the demonstrator let the door loose. In addition, only one piece of cat food was placed in the box for the demonstrator, preventing observers from scrounging.

### Group testing procedure

After the demonstrator had learned the task, we tested the entire group. We presented several boxes corresponding to the number of individuals in a group to avoid monopolisation of the apparatuses by the dominant female. The boxes were now baited with ten pieces of dry cat food to allow individuals to perform several trials repeatedly while both opening mechanisms were available. This time the door of the boxes closed automatically after the individual let the door loose, so that they had to open it again to obtain access to another reward. For the group testing procedure, each presentation of the apparatuses per group was considered as one session. Within a session, an individual could perform several trials. Subjects were tested until they reached a learning criterion of 80% of successful trials out of a minimum of 15 trials. Since several pieces of dry cat food were available, the apparatuses were not re-baited after each trial and were only removed after the last individual left the experimental arena.

### Video analyses

During the experiments, subjects were video-taped with a camcorder (SONY HDR-CX 240), and videos were analysed using Boris (Friard and Gamba 2016). From the demonstrator training sessions, we assessed social learning opportunities, which were defined as the time an observer spent together with a demonstrator within the metal ring, while the demonstrator was actively manipulating the boxes. To measure individual learning performance, we scored individual learning speed during the demonstrator training sessions and the group testing sessions. Learning speed was defined as the number of trials needed by an individual to reach the learning criterion, which was 80% of successful trials.

### Statistical analyses

We conducted all analyses using R statistical software (R Core Team 2017). First, to examine whether social learning opportunities co-varied with neophobia, we conducted Spearman’s correlation test between neophobia and social learning opportunities. Second, we examined whether the learning speed of observers in the demonstrator groups was predicted by neophobia and social learning opportunities by fitting a GLMM with a Poisson structure, using the package lme4 (Bates et al. 2014). Learning speed corresponds to the number of trials required by an individual to reach the learning criterion (80% of correct trials). Learning speed was fitted as the response variable and neophobia and social learning opportunities were fitted as fixed factors. We initially included the interaction term between fixed factors and we checked its significance using likelihood test ratio. When non-significant, the interaction between fixed factors was dropped from the analysis and the single terms were kept in the model. As the members of a group were present at the experimental arena during each test, we tested multiple individuals together at the same time. We, therefore, included group identity as random factor in the model.

Third, we examined whether learning speed was influenced by the presence of a demonstrator (yes or no). For this analysis, we used a Cox proportional hazards model with learning speed as the dependent variable, treating whether the individual learned the task or not (yes or no) as censored observations. The Cox model was conducted using the R-package “survival” (Therneau 2015).

Fourth, we examined whether observers in the pull and slide groups differed in the proportion of trials during which they used the pull technique using a Mann–Whitney *U* test. We also performed exact binomial tests to examine whether individuals in both the demonstrator and control groups developed a preference for one technique to solve the two-option task. We defined a preference when individuals used one technique more often than expected by chance to solve the two-option task. Moreover, using binomial tests, we examined whether the number of individuals that developed a preference differed from those that did not develop a preference for all groups and in the demonstrator groups only.

For the mixed models, we checked for collinearity between the fixed factors prior to all analyses. For all models, we performed likelihood test ratio for the full-null model comparisons and we visually inspected normality and homoscedasticity with residual plots. For the Cox proportional hazards model, we checked for the violation of proportional hazards.

## Results

### Neophobia

The latency to feed next to the novel object was significantly repeatable over time (*R* = 0.439, *p* = 0.04), whereas the latency to enter the ring (*R* = 0.001, *p* = 0.149) and the latency to contact the novel object (*R* = 0.073, *p* = 0.44) were not repeatable. Hence, we consider the latency to feed next to the novel object as neophobia. Neophobia did not correlate with social learning opportunities however (Spearman’s rank correlation test: *r* = 0.009, *p* = 0.989).

### Use of social information for learning

During the demonstrator training sessions, all five demonstrators learned to open the feeding apparatus. Four out of five demonstrators required 20 trials to learn the task, whereas one demonstrator (from group N) needed 28 trials. On average, the demonstrators needed 21.6 ± 3.6 (mean ± SD) trials to learn the task. Demonstrators needed on average 5.24 s (median, IQR: 4.77, *N* = 3) to open the door using the pull technique and 4.42 s (median, IQR: 5.27, *N* = 2) using the slide technique, suggesting that both techniques were equally difficult. During the group testing sessions, five out of seven observers in the pull groups and three out of four observers in the slide groups learned the task within 31 ± 16 (mean ± SD) trials. In the control groups, four individuals participated, but only one individual learned the task after 55 trials.

**Fig. 2:**
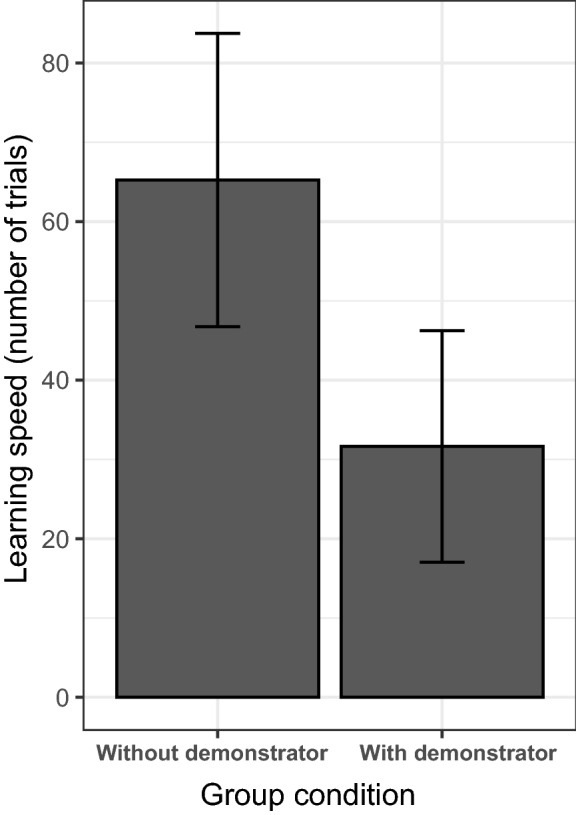
Difference in learning speed between individuals provided with demonstrators (pull and slide groups) and individuals without demonstrators (control groups): individuals in groups with a demonstrator learned the task faster

**Fig.3:**
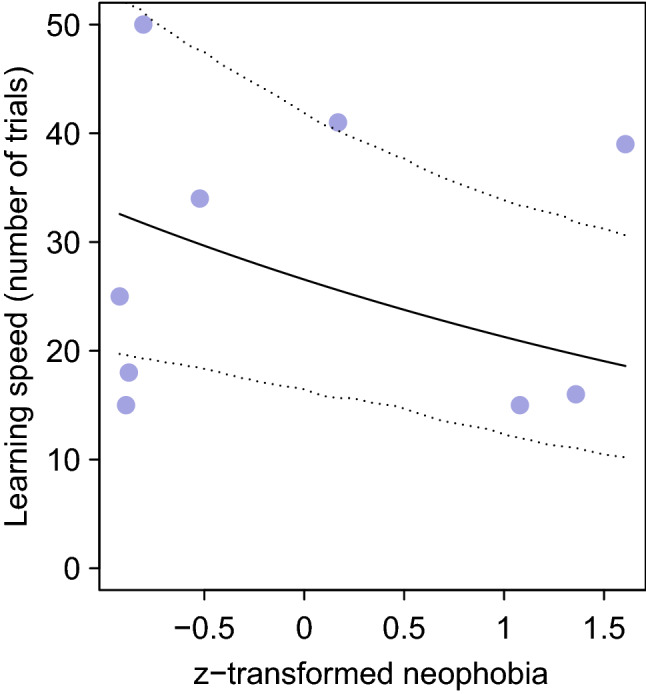
Influence of neophobia on individual learning speed during the testing sessions. Less neophobic individuals learned the task faster. The X-axis depicts the latency to feed close to a novel object as proxy of neophobia, with negative values representing less neophobic individuals and positive values for the more neophobic ones. Each circle represents an individual that learned the discrimination learning task. The continuous line depicts the fitted model, and the dotted lines depict its bootstrapped 95% confidence intervals

**Fig. 4:**
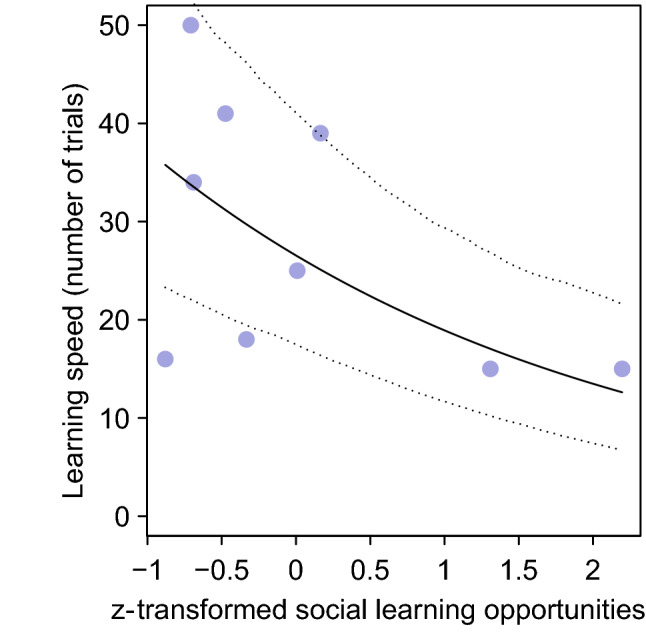
Influence of the tendency to seek social information on learning speed. Individuals who spent more time with the demonstrators within the metal ring solved the task faster. Each circle represents an individual that learned the discrimination learning task. The continuous line depicts the fitted model, and the dotted lines depict its bootstrapped 95% confidence intervals

The learning speed of observers co-varied with the presence of a demonstrator (*p* = 0.037, Table 2), with individuals in demonstrator groups learning the task faster compared to individuals in control groups (Fig. 2). During the demonstrator training sessions, observers spent on average 6.7 ± 13.7 min (mean ± SD) within the metal ring together with the demonstrator, our measure of social learning opportunities. We found that learning speed was influenced by both neophobia (*p* = 0.011; Table 3a) and social learning opportunities (*p* < 0.001; Table 3a), with less neophobic individuals and individuals seeking social learning opportunities for longer learning faster. Since one observer had a much longer latency to feed next to the novel object compared to the other individuals, we repeated the model without this outlier and obtained similar results for both neophobia (*p* = 0.022; Fig. 3; Table 3b) and social learning opportunities (*p* < 0.001; Fig. 4; Table 3b).

**Table 2:** Result of the Cox’s proportional hazards model assessing the effect of the presence of the demonstrator on individuals’ learning performances (*N* = 15)

Model	Fixed effect	Censor variable	*B* ± SE	*Z*	e (95% CI)	*P* value	Test for the proportional hazards
Effect of the presence of demonstrator on learning speed	Learning speed	Learning (yes or no)	2.32 ± 1.11	2.08	10.23 (1.15; 90.8)	**0.0369**	0.486

*B* beta coefficient*; SE* standard error; *z* Wald statistic value; *P p value*; e: exponentially transformed parameter estimates show the proportional change of hazard ratio, that is, the probability of solving the task, in response to unit change of predictors.; *CI* confidence interval of the hazard ratio Bold value indicates statistically significant result at the significance threshold *p* < 0.05

**Table 3:** Parameter estimates from the generalised linear mixed models (GLMMs) assessing the influence of neophobia and social learning opportunities on the learning speed of the observers, with corresponding full-null-model comparison

Variables		Parameter estimates					Likelihood ratio tests: full-null model comparisons				
Response variable	Fixed factors	Estimate	SE	*z*		*P*	*χ*^2^		df		*P*
[a]. Learning speed during the group testing sessions (*N* = 11)											
Number of trials until learning criterion	Intercept	3.304	0.163	20.207	< 0.001			27.432	2	< 0.001	
	z-transformed neophobia	0.161	0.063	2.526	0.011						
	z-transformed social learning opportunities	− 0.351	0.076	− 4.583	< 0.001						
[b]. Learning speed during the group testing sessions without outlier (*N* = 10)											
Number of trials until learning criterion	Intercept	3.279	0.227	14.435	< 0.001			29.732	2	< 0.001	
	z-transformed neophobia	− 0.221	0.097	− 2.281	0.022						
	z-transformed social learning opportunities	− 0.338	0.080	− 4.187	< 0.001						

*SE* standard error, *z* z-statistic value, *P p value*, *χ*^*2*^: *df*: degrees of freedom

Bold values indicate statistically significant results at the significance threshold *p* < 0.05

**Fig. 5:**
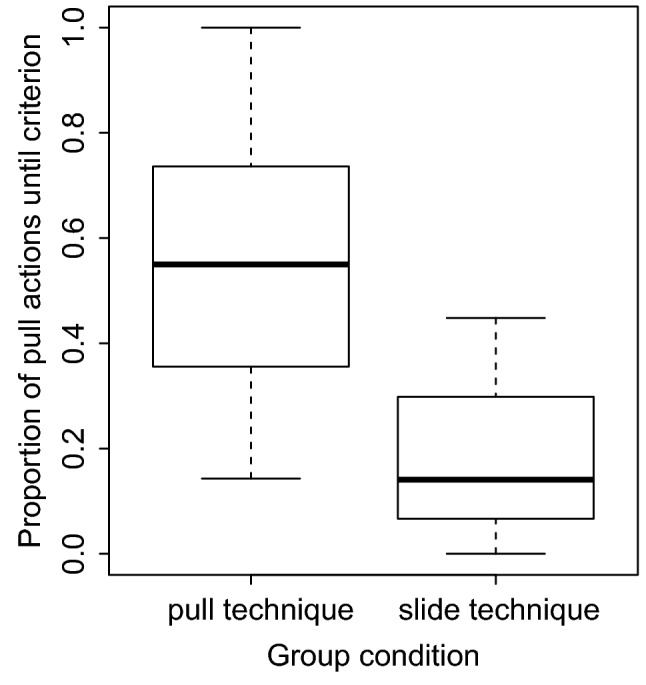
Proportion of pull actions made by individuals in the pull and slide groups. Individuals in the pull condition performed the pull actions more often compared to individuals in the slide groups

We also found that the proportion trials in which they used the pull technique differed between pull and slide groups, with observers in pull groups using the pull technique more often whereas observers in the slide groups used the pull technique less often and, hence, the slide technique more often (Mann Whitney *U* test, *W* = 25, *p* = 0.042, Fig. 5). All demonstrators maintained the technique learned during the group testing sessions, although three out of five demonstrators also discovered the other technique (Table 4). Overall, independent of whether individuals reached the learning criterion or not, eight individuals developed a preference for one technique and six exhibited no preference (binomial test, *p* = 0.795, Table 4). In the demonstrator groups, seven individuals developed a preference, whereas only one developed a preference in the control groups (*N* = 1, one-tailed binomial test, *p* = 0.035). However, from the seven individuals that developed a preference, five preferred the demonstrated technique whereas two individuals exhibited a preference for the other technique (one-tailed binomial test, *p* = 0.227).

**Table 4:** Results of the exact binomial tests to assess preferences for sliding or pulling techniques for each tested individual

Group	ID	Role	Learner	Pull actions performed	Slide actions performed	*p* value
Groups assigned to the pull condition						
G1	44	Demonstrator	–	14	1	< 0.001
	97	Observer	No	8	1	0.03
C	76	Demonstrator	–	13	0	< 0.001
	15	Observer	Yes	11	33	0.001
	22	Observer	Yes	22	18	0.635
	86	Observer	Yes	7	5	0.774
	87	Observer	No	4	24	< 0.001
N	95	Demonstrator	–	14	0	< 0.001
	94	Observer	Yes	6	7	1
	99	Observer	Yes	20	0	< 0.001
Groups assigned to the slide condition						
M	40	Demonstrator	–	2	11	0.02
	81	Observer	Yes	4	26	< 0.001
	89	Observer	Yes	4	23	< 0.001
	90	Observer	Yes	0	12	< 0.001
L1	75	Demonstrator	–	2	18	< 0.001
	96	Observer	No	13	16	0.711
Groups assigned to the control condition						
B	13	–	No	11	21	0.11
	66	–	Yes	18	26	0.291
	88	–	No	19	46	0.001
L	77	–	No	16	17	1

Bold values indicate statistically significant results at the significance threshold *p* < 0.5

## Discussion

We investigated the effect of neophobia on social learning in wild narrow-striped mongooses. Neophobia did not correlate with the propensity to seek social information. Less neophobic individuals and those that spent more time with the demonstrators during the demonstrator’s training sessions learned the task faster, suggesting that personality and social facilitation enhanced learning. The improvement of learning performance in the presence of a demonstrator indicates that local or stimulus enhancement may have fostered the acquisition of the different opening techniques. Moreover, the proportion of animals using the pull technique differed between observers in the pull and slide groups, indicating that individuals in the pull groups used the demonstrated technique more often, and five out of seven individuals developed a preference for the demonstrated technique. Hence, narrow-striped mongooses appear to rely on inadvertent social learning processes, such as social facilitation and local or stimulus enhancement, to deal with new challenges, such as the artificial feeding boxes.

In contrast to other species (Kurvers et al. 2010b), neophobia did not co-vary with the observers’ propensity to seek social information during the training sessions. However, both, neophobia and the tendency to seek social information influenced learning speed. Less neophobic individuals learned faster, supporting the hypothesis that fast personality types learn faster than slow personality types in a new situation (Sih and Del Giudice 2012). Moreover, the tendency to seek social information varied across individuals, indicating an attendance bias, which has been recognised to indicate directed social learning or transmission bias (Kendal et al. 2015). Narrow-striped mongooses that were more likely to seek social information also learned the task faster. Since the demonstrator was the dominant female of each group, the attendance bias might result from social inhibition by dominant females, constraining some observer’s tendency to approach her closely to avoid aggression (Schneider and Kappeler 2016).

Similarly, in meerkats and chimpanzees (*Pan troglodytes*), the rank of demonstrators influenced the tendency of lower-ranking individuals to seek social information during social learning (meerkats: Thornton and Malapert 2009; chimpanzees: Watson et al. 2017). Moreover, in Amazonian parrots (*Amazonia amazonica*), individuals receiving aggression at an artificial feeding apparatus interacted less often with the apparatus, thereby constraining their social learning opportunities (Morales Picard et al. 2017). In chacma baboons (*Papio ursinus*), boldness/neophobia did not co-vary with the tendency to pay attention to a demonstrator in a social learning experiment (Carter et al. 2014). As in our study, less neophobic individuals had greater learning success, but the tendency to seek social information did not influence learning success in baboons. Since the social learning task was relatively easy to solve, chacma baboons probably did not need much social information to solve the task, which benefitted bolder individuals that were more likely to interact with the novel food or feeding apparatus in solving the task faster (Carter et al. 2014). Hence, personality, but also the use of social information, can influence learning strategies when individuals are confronted with a new challenge.

Similarly, as several other species, such as red-fronted lemurs (*Eulemur rufifrons*; Schnoell and Fichtel 2012*),* Amazonian parrots (Morales et al. 2017) or blue tits (*Cyanistes caeruleus*; Aplin et al. 2013), narrow-striped mongooses in demonstrator groups learned the task faster than those in groups without a demonstrator, suggesting that the presence of a knowledgeable individual may have facilitated learning via local or stimulus enhancement (Hoppit and Laland 2008).

Narrow-striped mongooses also discovered the alternative technique to open the box. Individuals belonging to the demonstrator groups performed the demonstrated technique more often than the other one. Our findings here echo results from previous studies in which observers tended to adopt the technique displayed by the demonstrators or knowledgeable individuals (meerkats: Thornton and Malapert 2009, banded mongooses: Müller and Cant 2010, red-fronted lemurs: Schnoell and Fichtel 2012, and vervet monkeys: van de Waal et al. 2013). However, in the pull groups, only 2 out of 4 individuals that developed a preference preferred the pull technique. In the slide groups, all individuals that developed a preference, preferred the demonstrated slide technique. Hence, despite the fact that observers in the demonstrator groups used the demonstrated technique more often, not all individuals developed a preference for the demonstrated technique.

To summarize, we found that neophobia but also social information influenced problem-solving abilities in narrow-striped mongooses. Less neophobic individuals and those that tended to seek social information learned the task faster and the presence of a demonstrator facilitated learning, indicating the use of inadvertent social learning strategies, such as social facilitation and local or stimulus enhancement, to solve problems. Hence, our results emphasize the importance of also considering personality traits to obtain a more comprehensive view of social learning strategies. Finally, similar to other mongooses (Thornton and Malapert 2009), narrow-striped mongooses rely on the use of social information to solve problems, informing our understanding of social learning among carnivorans with different social systems.
